# Supplementary material for: Magnetoelectric coupling of domains, domain walls and vortices in a multiferroic with independent magnetic and electric order
Source: Nat Commun. 2021 May 25;12:3093. doi: 10.1038/s41467-021-22587-1 (PMC8149668; doi:10.1038/s41467-021-22587-1)
Supplement: Supplementary file 1 — Supplementary Information [file 41467_2021_22587_MOESM1_ESM.pdf]

Supplementary Information: Magnetoelectric coupling of domains, domain walls and vortices in a multiferroic with independent magnetic and electric order

Marcela Giraldo*, Quintin N. Meier*, Amadé Bortis, Dominik Nowak, Nicola A. Spaldin, Manfred Fiebig, Mads C. Weber†, Thomas Lottermoser† — *equal contributions — † equal supervision —

[1] *Department of Materials, ETH Zurich, 8093 Zurich, Switzerland.*

1 Second harmonic generation in $ErMnO_3$

Optical second harmonic generation (SHG) in the family of hexagonal manganites, h-*R*MnO_3, has been extensively studied in experiment and theory and is well understood [1–5]. For the convenience of the reader, we give a summary of the aspects relevant for the understanding of the present work.

Below the ferroelectric Curie temperature T_{C}, the crystallographic space-group of $ErMnO_3$ is $P6_3cm$. When $ErMnO_3$ orders antiferromagnetically at the Néel temperature T_{N}, the symmetry is reduced to $P6_3'cm'$. The ferroelectric order allows a nonlinear second-rank susceptibility tensor $\hat{\chi} \propto (\mathcal{P})$, while the antiferromagnetic order allows an additional tensor $\hat{\chi} \propto (\mathcal{PL})$ [4,6,7]. Its non-zero components are summarized in Table 1. The two susceptibilities give rise to electric-dipole contributions to the source term $\vec{S}(2\omega)$ of the emitted nonlinear light wave. An electric-dipole contribution proportional to a susceptibility tensor $\hat{\chi} \propto (\mathcal{L})$ is symmetry-forbidden. The lowest

order term is of the magnetic dipole type, but in experiments, a component of this type has not been detected [6].

Table S1: **Allowed SHG tensor components of $ErMnO_3$.** Triplets ijk in column 3 stand for the susceptibility χ_{ijk}.

	Space group	Tensor components χ_{ijk}
**Crystallographic SHG:** $\chi \propto \mathcal{P}$	$P6_3cm$	$xxz = xzx = yyz = yzy, zxx = zyy, zzz$
**Magnetic SHG:** $\chi \propto \mathcal{PL}$	$P6_3'cm'$	$yxx = xyx = xxy = -xxx$

Nevertheless, the sign of the purely antiferromagnetic order expressed by $\mathcal{L}$ is experimentally accessible with SHG. It is revealed by the interference of the crystallographic and magnetic SHG source terms $S_z(\mathcal{P})$ and S_y $(\mathcal{PL})$:

$$\begin{aligned} I(\mathcal{L}) &= |S_z(\mathcal{P}) + S_y(\mathcal{PL})|^2 \\ &\propto [(\chi_{zxx}(\mathcal{P})\sin\theta)^2 + (\chi_{yxx}(\mathcal{PL})\cos\theta)^2 - 2\chi_{zxx}(\mathcal{P})\chi_{yxx}(\mathcal{PL})\sin\theta\cos\theta\cos\Delta]E_xE_x \end{aligned} \tag{S1}$$

Here, the angle θ describes the rotation of the sample around the crystallographic x axis, and Δ is the phase between the two SHG contributions. As described in detail in the Methods section, the value of Δ, and, therefore, the sign of the interference term, are solely determined by the sign of the antiferromagnetic order parameter $\mathcal{L}$.

In Table 2 we summarized the measurement configurations which provide selective access to the

SHG source term components S_i proportional to $\mathcal{P}$, $\mathcal{PL}$ and $\mathcal{L}$. Deploying these configurations, we directly image the spatial distributions of the ferroelectric, antiferromagnetic and multiferroic hyperdomains.

Table S2: **Measurement configurations to reveal SHG contributions proportional to $\mathcal{P}$, $\mathcal{PL}$ and $\mathcal{L}$.** Sample is tilted by an angle θ around a crystallographic x axis (see Fig. S1). Incoming fundamental light is polarized parallel to the x axis, while the detected light is polarized perpendicular to the x axis revealing the y and z components of the respective SHG contributions.

**Temperature (T)**	**Tilt angle**	**SHG source term**	**order-parameter sensitivity**
$T_\mathrm{N} < T < T_\mathrm{C}$	$> 0°$	$S_z(\mathcal{P}) \propto \chi_{zxx}(\mathcal{P})E_xE_x \sin\theta$	$\mathcal{P}$
$T < T_\mathrm{N}$	$0°$	$S_y(\mathcal{PL}) \propto \chi_{yxx}(\mathcal{PL})E_xE_x \cos\theta$	$\mathcal{PL}$
$T < T_\mathrm{N}$	$> 0°$	$S_z(\mathcal{P}) + S_y(\mathcal{PL})$	$\mathcal{L}$

Figure S1 shows the SHG spectra of $ErMnO_3$ as a function of SHG energy for $S_z(\mathcal{P})$ and $S_y(\mathcal{PL})$ using a Coherent Ellite Duo laser system pumping an optical parametric amplifier for the spectral sweeps. The spectrum of the $S_y(\mathcal{PL})$ contribution shows a dip between 2.4 to 2.5 eV which is characteristic for $ErMnO_3$ [1,3].

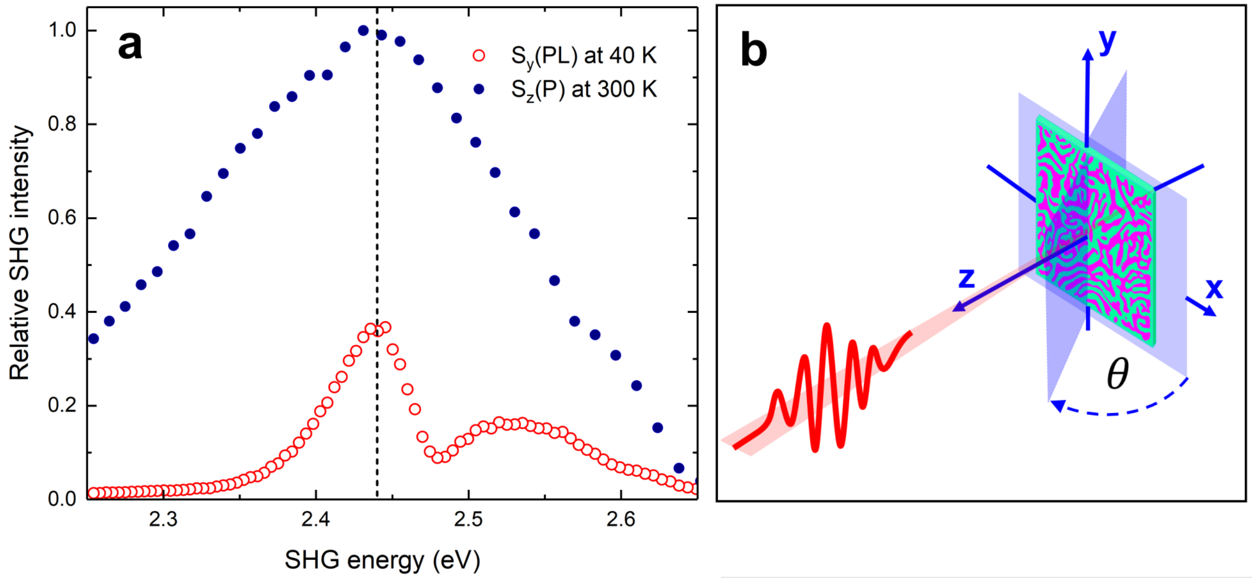

Figure S1: **Relative SHG intensity as a function of SHG energy.** **a**, Spectral dependence and relation between $S_z(\mathcal{P})$ at $\theta = 20°$ and $S_y(\mathcal{PL})$ at $\theta = 0°$, in agreement with refs 1, 3. The dashed line at 2.46 eV marks the position at which the SHG images in Figs. 2-3 and Figs. S2-S3 in this work were measured. **b**, Sketch depicting the direction of the incident fundamental light beam with respect to the z axis of the crystal. The rotated ($\theta > 0°$) and non-rotated ($\theta = 0°$) configurations allow to disentangle the magnetic and non-magnetic (crystallographic) SHG contributions, respectively.

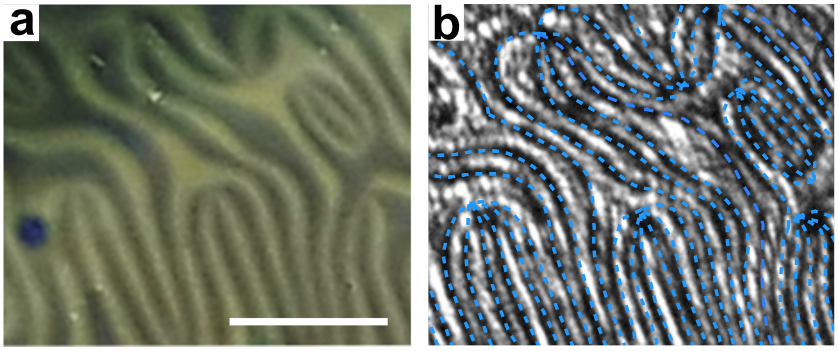

Figure S2: **Identification of ferroelectric $\pm\mathcal{P}$ domains in the SHG images.** **a**, Phase contrast microscopy image used to retrieve the position of the ferroelectric domain walls in Fig. 2d. Dark and bright regions correspond to opposite $\pm\mathcal{P}$ domains, which become visible because of the phase differences due to surface etching during the polishing procedure. **b**, Reproduction of Fig. 2d, showing the spatially resolved distribution of SHG intensity on the area of panel (a). Black lines (highlighted with dashed blue lines) indicate the position of the ferroelectric domain walls because of an SHG interference effect (see Methods section). Domains with $+\mathcal{P}$ and $-\mathcal{P}$ exhibit the same SHG brightness. Comparison with the phase contrast microscopy image in (a) allows distinguishing the $\pm\mathcal{P}$ domains. Images were taken at room temperature. Scale bar in (a) is 25 μm.

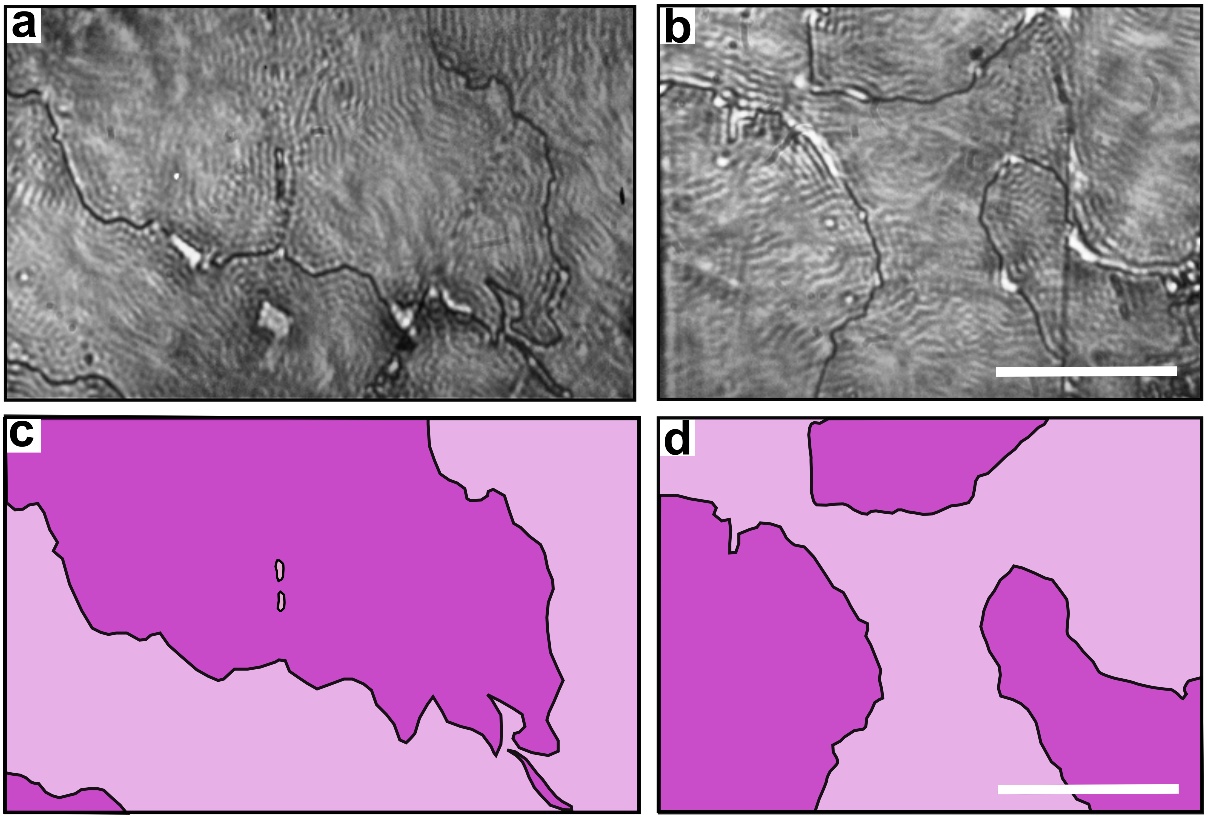

Figure S3: **Spatially resolved distribution of $\mathcal{PL}$ hyperdomains. a**, **b** SHG images of $\pm\mathcal{PL}$ hyperdomains on different regions of a c-oriented $ErMnO_3$ sample. Black lines indicate the position of the $\pm\mathcal{PL}$ hyperdomain walls (see Methods section). The hyperdomains are at least one order of magnitude larger in area than the antiferromagnetic domains shown in Figs. 2e and 3a-d. Both SHG images were acquired at 10 K after consecutive thermal annealing cycles through T_{N}. **c**, **d** Sketches of the the distribution of $\mathcal{PL}$ hyperdomains extracted from (a) and (b). Scale bar is 80 μm.

1. Fiebig, M., Fröhlich, D., Kohn, K., Leute, S., Lottermoser, T., Pavlov, V. V. & Pisarev, R. V. Determination of the magnetic symmetry of hexagonal manganites by second harmonic generation. *Phys. Rev. Lett.* **84**, 5620–5623 (2000).

2. Lottermoser, T., Fiebig, M. & Fröhlich, D. Symmetry and coupling of magnetic and electric order parameters in $YMnO_3$. *J. Appl. Phys.* **91**, 8251–8253 (2002).

3. Fiebig, M., Pavlov, V. V. & Pisarev, R. V. Second-harmonic generation as a tool for studying electronic and magnetic structures of crystals: review. *J. Opt. Soc. Am. B* **22**, 96–118 (2005).

4. Sa, D., Valentí, R. & Gros, C. A generalized Ginzburg-Landau approach to second harmonic generation. *Eur. Phys. J. B* **14**, 301–305 (2000).

5. Hanamura, E., Hagita, K. & Tanabe, Y. Clamping of ferroelectric and antiferromagnetic order parameters of $YMnO_3$. *J. Phys.: Condens. Matter* **15**, L103–L109 (2003).

6. Fiebig, M., Lottermoser, T., Fröhlich, D., Goltsev, A. V. & Pisarev, R. V. Observation of coupled magnetic and electric domains. *Nature* **419**, 818–820 (2002).

7. Lottermoser, T. *Elektrische und Magnetische Ordnung Hexagonaler Manganite.* (Ph.D. Thesis, Dortmund, 2002).
